# Postoperative interictal epileptiform discharges predict seizure recurrence after antiepileptic drug withdrawal regardless of concordance with surgical site

**DOI:** 10.1136/wjps-2023-000641

**Published:** 2024-02-17

**Authors:** Yuxin Wu, ZaiYu Zhang, Ping Liang, Lusheng Li, Bin Zou, Difei Wang, Xinyu Dong, Haotian Tang, Hanli Qiu, Xuan Zhai

**Affiliations:** Department of Neurosurgery, Children’s Hospital of Chongqing Medical University, National Clinical Research Center for Child Health and Disorders, Ministry of Education Key Laboratory of Child Development and Disorders, Chongqing, China

**Keywords:** Drug Withdrawal, Neurosurgery, Pediatrics

## Abstract

**Objective:**

The study aimed to explore the association between the site of interictal epileptic discharges (IEDs) on postoperative electroencephalogram (EEG) and seizure recurrence after antiepileptic drug (AED) withdrawal. The study hypothesizes that the concordance of IED sites with surgical sites indicates incomplete resection of epileptic focus, while non-concordance of IED sites with surgical sites indicates postoperative changes or cortical stimulation. The former has a higher risk of seizure recurrence.

**Methods:**

We retrospectively analyzed the postoperative EEG pattern of 182 consecutive children who underwent resection surgery. To identify the risk factors for seizure recurrence, we compared the attributes of seizure recurred and seizure-free groups by univariate and multivariate analyses. AED tapering was standardized, involving a 25% reduction in the dose of a single type of AED every 2 weeks, independent of the presurgical AED load.

**Results:**

We attempted AED withdrawal in 116 (63.7%) children. Twenty-eight (24.1%) children experienced seizure recurrence during or after AED withdrawal. A greater number of AEDs used at the time of surgery (*p*=0.005), incomplete resection (*p*=0.001), and presence of IED on postoperative EEG (*p*=0.011) are predictors of seizure recurrence. The completeness of resection and seizure recurrence after AED withdrawal were related to the presence of IED on the EEG, but not to the concordance of IED with surgical sites.

**Conclusion:**

For children with abnormal EEG, the decision to discontinue AED should be made more cautiously, regardless of the relative location of the discharge site and the surgical site.

**WHAT IS ALREADY KNOWN ON THIS TOPIC**

- The presence of interictal epileptic discharges (IEDs) on scalp electroencephalogram (EEG) is an accepted risk factor for seizure recurrence after antiepileptic drug (AED) withdrawal.
- This made IEDs a potential indicator for continuing AED therapy, with a standard procedure being AED withdrawal in the presence of a normal EEG combined with a period of seizure freedom.
- Previous studies have focused on the presence of IED or the discharge patterns and ignored the sites of IED.

**WHAT THIS STUDY ADDS**

- This research reveals that while IED presence postoperatively may indicate a higher risk of seizure recurrence, its specific location, especially in relation to surgical sites, may not be as indicative as previously believed.
- Specifically, the completeness of resection and seizure recurrence after AED withdrawal were related to the presence of IED on the EEG, but not to the concordance of IED with surgical sites.

**HOW THIS STUDY MIGHT AFFECT RESEARCH, PRACTICE OR POLICY**

- In medical practice, there has been an oversight on how to interpret and respond to the presence of IED, with prior studies often neglecting the significance of the location of IED.
- This research provides a new direction on how to use EEG to guide strategies for discontinuation of AED after epilepsy surgery, prompting a re-evaluation of how EEG results are interpreted in postoperative care.
- This will assist in more accurately assessing and predicting the risk of seizure recurrence after AED withdrawal, leading to more informed treatment strategies.

## Introduction

Epileptic resection surgery is an effective treatment for intractable epilepsy, with an overall seizure-free rate of 65% at 1-year after surgery.^1^ A topic of concern after surgery is whether and when to start antiepileptic drug (AED) withdrawal.^2^ Due to significant adverse effect burden of AED,^3 4^ early AED withdrawal can improve the quality of life and neurocognitive functions, particularly of pediatric patients.^3 5 6^ A recent study investigated the opinions of 277 parents about epilepsy surgery in children with epilepsy and found that the most common reason for consent to epilepsy surgery was the possibility of AED withdrawal after surgery.^7^ However, AED withdrawal is not an easy decision, as children may experience seizure recurrence after AED withdrawal, which may create new problems (e.g., injuries and loss of self-esteem).^8^ There is currently no standardized procedure for AED withdrawal, and practices are highly variable between centers.^9^ A 2012 survey of 58 Canadian pediatric and adult epileptologists reported that there was substantial disagreement concerning the time to AED withdrawal: 10% epileptologists attempted AED withdrawal before 6 months after surgery, 21% attempted 6–11 months after surgery, and 50% attempted >1 year after surgery.^10^

To develop appropriate and individualized withdrawal procedure, previous literature has explored many potential risk factors for seizure recurrence. Interictal epileptic discharges (IEDs) on prediscontinuation electroencephalogram (EEG) have been identified as potentially associated with the risk of seizure recurrence after medication withdrawal.^11–14^ In a large meta-analysis focusing on patients with epilepsy who received only AED treatments (without surgery), EEG abnormalities prior to discontinuation were found to be an independent predictor of seizure recurrence.^15^ Hence, for children with IED on postoperative EEG, physicians tended to continue AED treatments.^10^ However, even though IED appeared to increase the risk of seizure recurrence, there were still a significant proportion of children with IED who still achieved seizure freedom after AED discontinuation.^16^

Considering that previous studies have focused on the presence of IED or the discharge patterns and ignored the sites of IED, this study was designed to explore the association between the sites of IED on the EEG and seizure recurrence. The present study was based on the hypothesis that the concordance of IED sites with surgical sites may indicate a residual epileptic focus. The inconsistency of IED sites with surgical sites may represent postoperative changes or cortical stimulation. The former may have a higher risk for potential relapse and this difference in IED sites was the reason why some children with IED on the EEG could achieve seizure freedom after AED withdrawal.

## Methods

### Patients

This retrospective study was conducted at the Department of Neurosurgery of Children’s Hospital of Chongqing Medical University (Chongqing, China), which is the only tertiary pediatric epilepsy medical center in Southwest China providing nationalized specialist service for childhood epilepsy surgery. Written informed consent was obtained from each participant. Children who underwent epilepsy surgery from January 2014 to January 2021 were included according to the following criteria: (1) age at operation <18; (2) lesion resection, lobectomy, or multilobar resection surgery was performed; and (3) were followed up for more than 1 year after surgery and more than 6 months after AED withdrawal. The exclusion criteria were as follows: (1) underwent surgery or hemispherectomy; (2) with bilateral or diffuse epileptogenic focus; (3) surgical resection site mainly involving the medial cortex, such as medial temporal lobe epilepsy and insular epilepsy; (4) underwent surgery for hypothalamic hamartoma; and (5) lack of postoperative EEG data. Epileptic foci in deep or mesial locations may produce extensive, non-localizing epileptiform discharges, resulting in lower spatial resolution in EEG.^17^ This study focused on the association between the site of IED and seizure recurrence after AED withdrawal so as to avoid bias due to inaccurate IED localizing. Children with epileptic foci in deep or mesial location, such as hypothalamic hamartoma, were excluded from the study cohort. Lobectomy, multilobar resection surgery, and hemispherectomy may also have an enormous impact on IED localizing due to anatomical removal of a lobe of the brain. For example, IEDs are unlikely to appear in the temporal lobe after temporal lobectomy due to the anatomical absence of temporal lobe. Although this concern was not substantiated, we carefully excluded them from the analysis cohort. For children who underwent two or more epilepsy surgeries, the outcomes of the first surgery were used for analysis.

### Patient and public involvement

Patients and/or the public were not directly involved in the design, conduct, reporting, or dissemination plans of this research.

### Preoperative evaluations

All patients underwent standard presurgical evaluations, including seizure history, neurological examination, high-resolution brain MRI with a 3T epilepsy protocol, and video EEG according to the 10–20 system, at the multidisciplinary team (MDT) of epilepsy surgery of the Children’s Hospital of Chongqing Medical University. If non-invasive methods did not yield consistent findings with a single resectable epileptogenic lesion or if the potential epileptogenic zone involved highly eloquent cortex, then fluorodeoxyglucose positron emission tomography and invasive electroencephalography (electrocorticography or stereoelectroencephalography) were performed. The type of surgery and the extent of resection were decided together by the MDT based on the presumed epileptogenic zones. Complete resection of the assumed epileptic zone was defined by resection of the visible lesion on MRI and the ictal onset zone that was confirmed during presurgical evaluation. In collaboration with experienced radiologists, neurosurgeons initially classified lesion resection status by comparing the preoperative and postoperative MRIs of the abnormal structures into categories: complete resection, incomplete resection, and undetermined (when preoperative MRI was negative). Subsequently, for patients with negative preoperative MRIs, the completeness of the resection was further determined based on whether all areas identified as the epileptogenic zone in the preoperative evaluation were removed.

### Postoperative management

All children underwent postoperative follow-up every 3 months, during which scalp EEGs were conducted at each visit. The prerequisite for initiating AED withdrawal was that the child had to be seizure-free for at least 6 months postsurgery and display no IED on two consecutive scalp EEGs. In this study, spikes, sharp waves, paroxysmal slowing, or non-paroxysmal abnormalities were all defined as EEG abnormalities. Once a child met these criteria, we engaged in discussions with their caregivers regarding the potential benefits and risks of AED withdrawal. The withdrawal process commenced only after obtaining explicit consent from the family. The AED tapering protocol involved reducing the dosage of a single type of AED by 25% every 2 weeks. If the EEG exhibited IEDs during this tapering phase or if the child experienced seizure recurrence, the withdrawal would be halted immediately. Subsequently, AED treatments would be reintroduced or adjusted until the child achieved a seizure-free state. The tapering persisted until one of the following occurred: complete AED discontinuation, emergence of an abnormal EEG, or seizure recurrence. In the event of an abnormal EEG or seizure recurrence, the AED withdrawal was suspended immediately and AED treatments were reintroduced or optimized to regain a seizure-free status.

### EEG characteristics

A previous study found that EEG at 3 months after surgery cannot predict recurrence after AED discontinuation.^16^ It is possible that the duration of follow-up is too close to the time of operation, which makes it more sensitive to postoperative changes and cortical irritation, while IED on the EEG at 1 year after surgery can predict seizure recurrence after drug withdrawal.^16^ Since AED withdrawal was aggressive and some children attempted drug reduction as early as 6 months after surgery, we collected the EEG signals 6 months after surgery to analyze the relationship between IED and seizure recurrence after AED withdrawal.

All EEG data were evaluated by an experienced neurologist and an experienced EEG clinician. Spikes, sharp waves, paroxysmal slowing, or non-paroxysmal abnormalities were considered as IED. For analysis of the concordance of surgical sites and IED sites, we classified scalp EEG as “normal,” “localizing,” “lateralizing,” or “non-concordant,” with reference to a previous study. Normal was allocated to EEG without IED; localizing was used when IED was located in the same lobe or quadrant as the surgical sites; lateralizing was defined as IED keeping with the hemispheric lateralization of the surgical sites but could not further localize within the hemisphere; and non-concordant was defined as IED in keeping with neither the cortical area nor the hemisphere of the surgical sites.^18^ Localizing and lateralizing IEDs were combined as “concordant” with the surgical sites, and the seizure recurrence rates of children with concordant EEG were compared with those who had non-concordant EEG during or after AED withdrawal.

### Statistical analysis

SPSS software (SPSS for Windows V.23.0) was used for statistical analysis. Descriptive statistics were used for demographic data. Categorical variables were summarized as numbers (percentages), while continuous variables were presented as mean±standard deviation (SD). Children with versus without seizure recurrence during or after AED withdrawal were compared using χ^2^ or Fisher’s exact test for categorical data and Mann-Whitney U test for numeric data. Pairwise comparison in multiple categorical variables was conducted using the α segmentation method. P values were adjusted using the Benjamini-Hochberg correction. The risk factors investigated in the comparison subsequently subjected to multivariate logistic regression, and two sensitive analyses were performed. We did not modify the withdrawal protocol based on the type of AED used, instead implementing a relatively rapid tapering schedule for all patients. Although there is no definitive consensus on the optimal tapering rate for AEDs, we are aware that certain drugs (phenobarbitone, carbamazepine, nitrazepam, and clonazepam) may induce withdrawal seizures when tapered rapidly, potentially affecting the reliability of our results. With this in mind, we re-examined the association between IED concordance and seizure recurrence after excluding patients on AEDs with a known risk of withdrawal seizures. In addition, given the differences in IED localization capabilities in different brain lobes, we further divided the cohort into temporal lobe epilepsy and extratemporal epilepsy and examined the association between IED concordance and seizure recurrence in the two subgroups. The results are reported as hazard ratio (HR) with 95% confidence interval (CI). P values were based on two-sided tests, with 0.05 as the cut-off level for statistical significance. All data relevant to the study are included in the article or uploaded as online supplemental file 1.

10.1136/wjps-2023-000641.supp1 Supplementary data

## Results

### Patient characteristics

**Figure 1:**
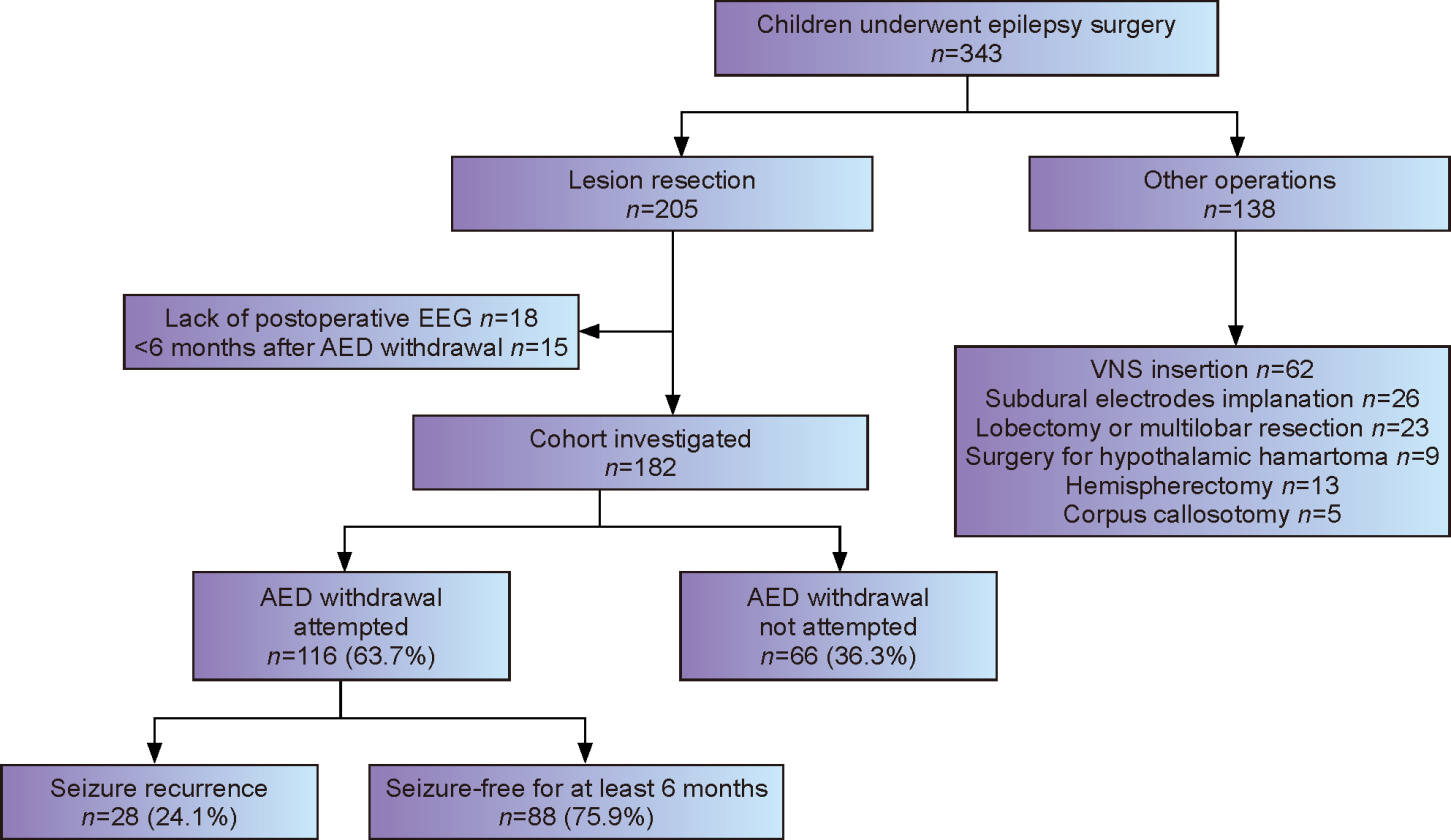
Flowchart of study population and longitudinal antiepileptic drug (AED) therapy outcomes. EEG, electrocorticography; VNS, vagus nerve stimulation; AED, antiepileptic drug.

A total of 182 children were included in the study, and the flow chart of patients included in this study is depicted in figure 1. A total of 116 patients (63.7%) attempted AED tapering 11.7±9.9 months after surgery and 105 (55.9%) attempted AED discontinuation 14.2±10.1 months after surgery. For the cohort of 116 children who attempted AED withdrawal (66 males, 50 females), the mean follow-up time was 45.3±18.0 months after surgery, 33.6±18.0) months after AED tapering, and 30.7±16.9 months after AED discontinuation. Pathologically, 45 patients had tumors as the most common abnormality, followed by focal cortical dysplasia (*n*=27) and vascular malformation(*n*=27). The characteristics of the children who attempted AED withdrawal are shown in table 1.

**Table 1:** Characteristics of children with epilepsy who attempted antiepileptic drug withdrawal

Characteristics	Patients (*n*=116) *n* (%)/mean±SD
Gender	
Male	66 (56.9)
Female	50 (43.1)
Age at epilepsy onset, months	60.2±39.3
Age at epilepsy operation, months	78.2±37.9
Epilepsy duration, months	18.0±29.9
Number of AEDs used at the time of surgery	1.2±0.4
Left-sided surgery	56 (48.3)
Lesion location	
Frontal	33 (28.4)
Parietal	23 (19.8)
Temporal	20 (17.2)
Occipital	11 (9.5)
Multilobar	29 (25.0)
Histopathological finding	
Tumor	45 (38.8)
FCD	27 (23.3)
Vascular malformation	27 (23.3)
Others*	17 (14.7)
Postoperative interictal EEG pattern	
Normal	87 (75.0)
Localizing	9 (7.8)
Lateralizing	6 (5.2)
Non-concordant	14 (12.1)
Time after surgery to AED withdrawal, months	11.7±9.9
Seizure recurrence during or after AED withdrawal	28 (24.1)
Complete resection	99 (85.3)

*Other histopathological findings: gray matter heterotopia: 3 children; encephalomalacia: 4 children; demyelinating disease: 4 children; tuberous sclerosis: 3 children; arachnoid cyst: 3 children.

AED, antiepileptic drug; EEG, electroencephalogram; FCD, focal cortical dysplasia.

### EEG pattern after surgery

**Figure 2:**
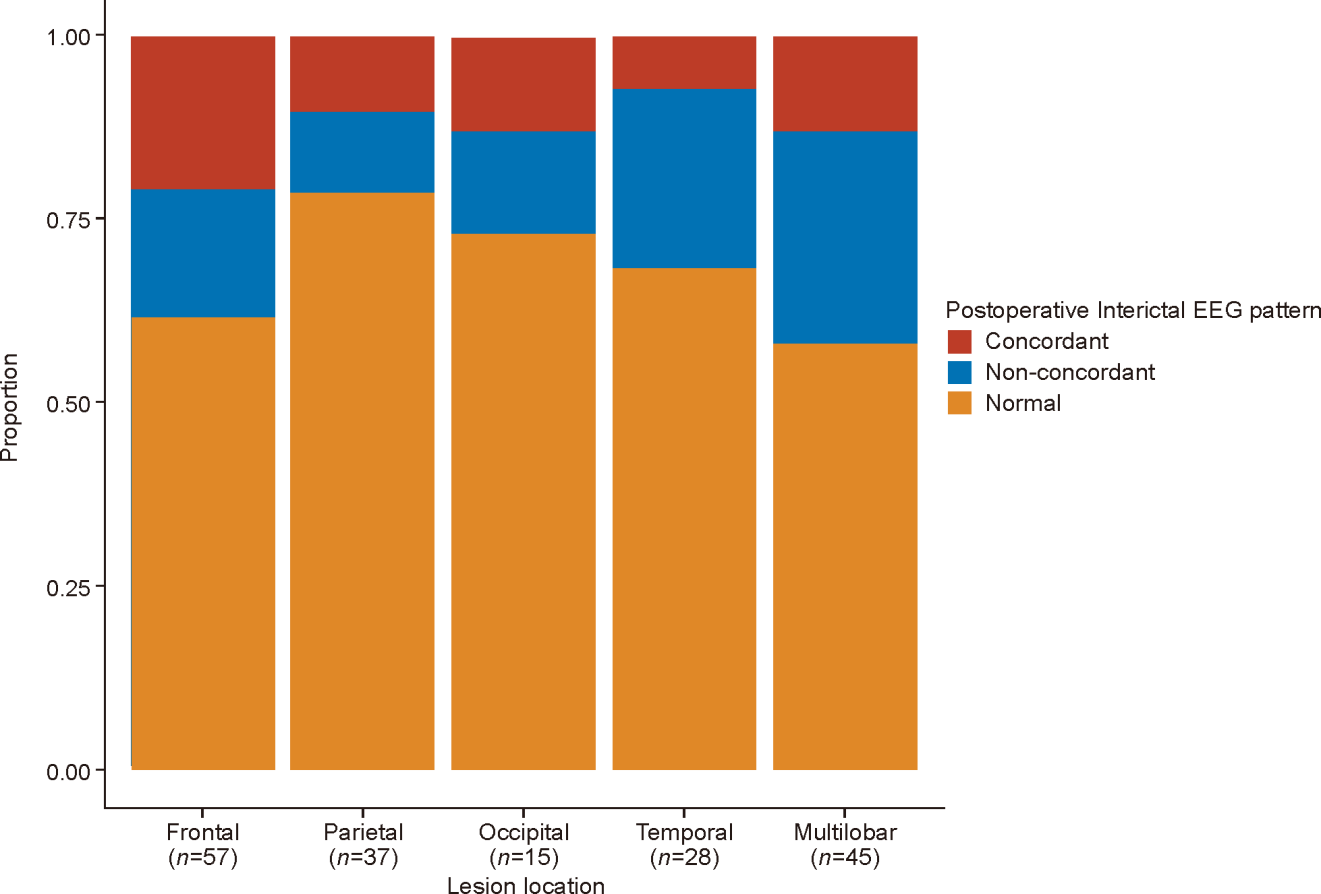
Postoperative Interictal EEG pattern in different brain lobes in children with epilepsy. EEG, electroencephalogram.

Among the 182 children who underwent resection surgery, 120 (65.9%) had a normal EEG 6 months after surgery. In 26 (14.3%) children, the IED sites and surgical sites were concordant, and in 36 (19.8%) children the IED sites and surgical sites were non-concordant. The percentage of complete resection in patients with IED on the EEG postoperatively was 69.4% (43 out of 62) compared with 83.3% (100 out of 120) of patients without IED, which was statistically different (*p*=0.029). While complete resection rates in concordant EEG and non-concordant EEG were 69.2% and 69.4%, the distribution in the two groups did not differ significantly (*p*=0.99). The completeness of resection of epileptic foci was related to the presence of IED on the EEG, but not to the concordance of IED sites with surgical sites. By comparing the distributions of scalp EEG patterns in different surgical brain lobes, no statistically significant differences were found (*p*=0.254). The scalp EEG patterns in different surgical brain lobes are shown in figure 2. For 116 children who attempted AED withdrawal, 87 (75.0%) had normal EEG, 15 (12.9%) had a concordant EEG, and 14 (12.1%) had a non-concordant EEG.

### Seizure recurrence

At the latest follow-up, 134 of 182 (73.6%) children achieved seizure freedom. Among them, 79 (43.4%) were seizure-free without any AED and 55 (30.2%) seizure-free with the help of at least one type of AED. Seizure recurred in 28 (24.1%) of the 116 children who attempted AED withdrawal. Eighteen children experienced seizure recurrence during AED tapering and 10 children experienced seizure recurrence after complete AED discontinuation. The cumulative recurrence rate increased progressively after initiating AED withdrawal: 16.4% (19 out of 116) at 6 months, 18.9% (20 out of 106) at 1 year, 25.9% (21 out of 81) at 2 years, and 25.7% (10 out of 39) at 3 years or more. Notably, 82.1% (23 out of 28) of patients experienced seizure recurrence within the first year after AED withdrawal. The mean time from AED withdrawal to seizure recurrence was 6.3±5.4 months. A comparison of potential predictors between children with and without recurrent seizures during or after AED withdrawal based on univariate comparisons is presented in table 2. Univariate comparisonsrevealed that the number of AEDs used at the time of surgery (*p*=0.003), postoperative interictal EEG pattern (*p*=0.003), and complete resection (*p*=0.003) were associated with seizure recurrence. In terms of the presurgical drug load, children were categorized into two groups: monotherapy and polytherapy. Among those on monotherapy, 15.7% (14 out of 89) had seizure recurrence, and 51.9% (14 out of 27) had recurrence during polytherapy. The difference between the two groups was statistically different (*p*<0.001). Seizure recurrence rates in children with normal EEG, concordant EEG, and non-concordant EEG were 14.9%, 53.3%, and 50.0%, respectively. The seizure recurrence rates between the normal EEG group and the concordant EEG (*p*=0.002) and non-concordant EEG (*p*=0.007) groups differed significantly, while the seizure recurrence rates between the concordant EEG and non-concordant EEG did not differ (*p*=1.00) (see table 3). In sensitivity analyses, we excluded patients taking AEDs known for their potential to induce withdrawal seizures (*n*=7) and the findings were consistent. The seizure recurrence rates significantly differed between patients with normal EEG and those with abnormal EEG (*p*=0.002). However, there was no significant difference in seizure recurrence rates between the concordant EEG and non-concordant EEG groups (*p*=1.000). In a separate sensitivity analysis, given the limited number of cases with temporal lobe lesions (only 20), the sample size was deemed insufficient for robust statistical analysis. Thus, our focus shifted exclusively to the extratemporal epilepsy group. Similarly, a significant correlation was noted between postoperative interictal EEG patterns and seizure recurrence following AED withdrawal (*p*<0.001). Nevertheless, the seizure recurrence rates did not significantly differ between the concordant EEG and non-concordant EEG groups (*p*=0.41). Multivariate logistic regression analysis found that the number of AEDs used at the time of surgery (*p*=0.005), complete resection (*p*=0.001), and postoperative interictal EEG pattern (*p*=0.011) are significant predictors of seizure recurrence (table 4). Children whose postoperative EEG showed IED had a greater risk of seizure recurrence than whose with normal EEG. The concordance of IED sites and surgical sites did not predict higher risk of seizure recurrence.

**Table 2:** Comparison of variables in seizure-free and seizure recurrence groups

Variables	Seizure-free (*n*=88) *n* (%)/mean±SD	Seizure recurrence (*n*=28) *n* (%)/mean±SD	*P* value	Adjusted *p* value*
Male	48 (54.5)	18 (64.3)	0.365†	0.608
Age at epilepsy onset, months	60.7±38.4	58.6±42.6	0.599‡	0.684
Age at epilepsy operation, months	76.7±36.6	82.9±42.3	0.521‡	0.684
Duration of epilepsy, months	16.0±27.7	24.4±35.7	0.066‡	0.132
Number of AEDs used at the time of surgery	1.2±0.4	1.5±0.5	<0.001‡	0.003
Time after surgery to AED withdrawal, months	12.2±10.6	9.8±7.2	0.700‡	0.700
Multilobar lesion	21 (23.9)	8 (28.6)	0.616†	0.684
Pathological findings			0.022†	0.055
Tumor	41 (46.6)	4 (14.3)		
FCD	17 (19.3)	10 (35.7)		
Vascular malformation	18 (20.5)	9 (32.1)		
Other	12 (13.6)	5 (17.9)		
Postoperative interictal EEG pattern¶			<0.001†	0.003
Complete resection	82 (93.2)	17 (60.7)	<0.001§	0.003

*P values were adjusted using the Benjamini-Hochberg correction.

†P value by χ^2^ test.

‡P value by Mann-Whitney U test.

§P value by Fisher’s exact test.

¶Pairwise comparison was conducted by α segmentation method (see table 3).

AED, antiepileptic drug; EEG, electroencephalogram; FCD, focal cortical dysplasia.

**Table 3:** Pairwise comparison between seizure recurrence rates in different electrocorticography patterns by α segmentation method

Variables	Seizure-free (*n*=88)	Seizure recurrence (*n*=28)	*P* value
Normal	74	13	0.002*
Concordant	7	8	
Normal	74	13	0.007*
Non-concordant	7	7	
Concordant	7	8	1.000
Non-concordant	7	7	

P<0.017 was considered statistically significant.

* indicates statistical significance.

**Table 4:** Predictors of seizure recurrence in children who attempted AED withdrawal by logistic regression analysis

Variables	Regression coefficient	SE of regression coefficient	OR (95% CI)	*P* value
Number of AEDs used at the time of surgery	1.627	0.600	5.1 (1.6 to 16.5)	0.005*
Complete resection	2.403	0.698	11.5 (2.8 to 43.4)	0.001*
Postoperative interictal EEG pattern	1.845	0.729	6.3 (1.5 to 26.4)	0.011*
Concordant vs normal	1.888	0.675	5.6 (1.8 to 24.8)	0.005
Non-concordant vs normal	1.845	0.729	6.3 (1.5 to 26.4)	0.011

* indicates statistical significance.

AED, antiepileptic drug; EEG, electroencephalogram.

## Discussion

The present study was designed to investigate the relation between the concordance of IED sites and surgical sites and seizure recurrence after AED withdrawal, and to explore other potential predictors of seizure recurrence. This cohort included 188 children who underwent resection with at least 6 months of follow-up after AED withdrawal. A total of 43.4% of children were seizure-free and AED-free after surgery, similar to previously published AED-free rates in children.^3^ Overall, 24.1% of children experienced seizure recurrence during or after AED withdrawal. A greater numbers of AEDs used at the time of surgery, presence of IED on postoperative EEG, and incomplete resection were associated with a greater likelihood of seizure recurrence during or after AED withdrawal.

Scalp EEG is a non-invasive examination that records the brain’s electrical activity with high temporal resolution, and is important in the diagnosis, treatment, and postoperative management of epilepsy.^2^ Previous studies on the relationship between postoperative EEG and seizure recurrence after AED withdrawal mostly focused on the presence of IED. A meta-analysis from 2019 outlined the relationship between EEG pattern and seizure recurrence after AED withdrawal in simple pharmacological treatment and indicated that the IED on scalp EEG during or after AED withdrawal is a risk factor for seizure recurrence.^19^ Similar findings have also been previously reported in a surgical cohort.^11–13 15^ Thus, in children with postoperative EEG epileptiform discharge, physicians usually prefer to maintain AED therapy. A normal EEG combined with a period of seizure freedom is one of the most common factors influencing doctors’ decisions to withdraw AED.^10^ On this basis, the present study explored the association between postoperative EEG pattern and seizure recurrence after AED withdrawal and hypothesized that the concordance of the discharge sites with the surgical sites may be due to residual epileptic focus, which represents a greater risk of seizure recurrence. We adopted a fairly strict criterion. Our results are in accordance with those of the present studies, indicating that the presence of IED on postoperative EEG is a predictor of seizure recurrence after AED withdrawal. However, no statistically significant correlation was observed between concordance of IED with surgical sites and seizure recurrence. Moreover, there was no significant difference in the complete resection rates between children with concordant and non-concordant EEG after surgery. Subsequent sensitive analyses, based on epileptogenic lesion location and type of AED, yielded consistent results. This is a rather disappointing result. We suspected that the differences in the ability of IED localizing in different brain lobes contributed to this outcome. A previous study of 390 patients with focal epilepsy compared the IED sites with the MRI sites and found that the temporal lobe IED was most consistent with surgical sites on MRI.^17^ We then divided the children into temporal lobe and extratemporal epilepsy groups, and conducted a subgroup analysis in the t two groups, but the results did not change.

We believe that this unexpected finding might be a result of the poor spatial resolution and signal to noise ratio of EEG.^20 21^ The propagation of EEG signals in the brain is constructed in by volumetric conduction. Volume conduction is the process by which a pool of ions repel nearby ions of the same charge.^22^ The brain is filled with dipoles and each dipole has a charge effect in all directions in space. Each dipole affects not only the charge of the scalp above the dipole, but the charge of the whole scalp. Thus, the voltage fluctuations measured by any electrode on the scalp are the result of the joint charge activity of multiple field potential sources, which is also called the spatial ambiguity effect of the EEG signal.^23^ Therefore, due to the poor spatial resolution of EEG signals, it is not appropriate to extract spatial information simply focusing on the consistency of the IED lobe with that of the surgical lobe. Future investigations could explore the benefits of integrating IED localization with other modalities such as functional MRI or magnetoencephalography, which provides more refined spatial information. These combined approaches could potentially improve our understanding of epileptic networks and guide postoperative AED treatment.

Another possible explanation for this finding is that the propagation of IED is spread. For example, many occipital seizures show temporal IED on the preoperative EEG. In temporal epilepsy, the IED may spread to the contralateral hippocampus rather than to the ipsilateral temporal neocortex.^17^ Because of these EEG characteristics, the value of the location of IED needs to be interpreted more carefully. Rather than simply comparing the lobe of IED and the surgical lobe, we need to explain the postoperative IED sites relying on the preoperative IED characteristics of different brain lobes or even different pathological lesions. This study did not find the reason why a large number of children with abnormal EEG could safely stop AEDs, but we insist that it makes sense to focus on the relationship between postoperative EEG and seizure recurrence. Although the presence of IED on postoperative EEG indicates a higher risk of seizure recurrence, half of the children with IED achieved seizure-free after AED withdrawal. Hence, an abnormal postoperative EEG is not an absolute contraindication to attempting AED tapering.^16^ It has been suggested that one of the preconditions to start AED withdrawal is a normal EEG, but it is not fair for children who have the potential for AED withdrawal. It is important to identify the reasons for the different seizure outcomes in children with abnormal EEG, which can be helpful in establishing a standard postoperative management protocol. This study preliminarily excluded the influence of concordance of the site of IED with the surgical site, and the reasons for the different seizure outcomes need to be further explored in subsequent studies.

In the present study, 23.5% of children experienced seizure recurrence during or after AED withdrawal, and the previously reported recurrence rate was 6%–35%.^13 14 24–27^ The recurrence rate in the present cohort was relatively close to the recurrence rates reported in previous studies. Previous studies have demonstrated that the risk of seizure recurrence after AED withdrawal was lower in children with more precisely localized low-grade tumors or vascular malformations compared with children with focal cortical dysplasia.^11^ However, in our study, there was no statistically significant difference in the likelihood of postwithdrawal seizure recurrence across different pathological substrates.

This study also found that a greater number of AEDs used at the time of surgery and incomplete resection were associated with a greater risk of seizure recurrence during or after AED withdrawal, which is in agreement with previous findings. It is reasonable to assume that epileptogenic regions remaining in children who underwent incomplete resection would increase the risk of seizure recurrence after AED withdrawal.^27^ High risk of seizure recurrence in children with more AEDs at the time of surgery may be attributable to our withdrawal process.

Radhakrishnan *et al* found in a cohort of adults undergoing temporal lobectomy that two-thirds of seizure recurrence related to AED withdrawal occurred during AED reduction and one-third occurred after complete AED discontinuation.^12^ Although their cohort attempted AED reduction early after surgery, the withdrawal process was quite cautious, taking close to 3 years from the AED tapering to complete discontinuation. The withdrawal process in our cohort was relatively aggressive, with a mean time taken from AED tapering to complete discontinuation of 3.6±4.2 months. The AEDs were typically reduced by 25% of the dose for a single AED type every two weeks until complete discontinuation of all drugs. In other centers, a period of observation usually followed the discontinuation of one type of AED. Interestingly, although the duration of AED reduction in our cohort was very short, two-thirds of seizures in our cohort still occurred during the drug reduction period. The children in out cohort were followed up for 30.6±16.7 months after complete drug reduction, but only one-third of the seizure recurrence occurred during this period. It seems reasonable to suppose that there is a pathophysiological basis for children experiencing seizure recurrence after AED withdrawal. They appear seizure-free under the guise of antiepileptic medication. Once the AEDs were withdrawn, they have a high probability of seizure recurrence. A 2011 TTS(time to stop) study including 766 children found that early AED withdrawal does not affect long-term seizure outcome.^25^ The present study supports that neither the timing of initiation of AED withdrawal nor the speed of AED withdrawal affects long-term seizure outcome; a shorter withdrawal process may earlier reveal the “not entirely successful surgery.” The importance of the observation period is unclear, and future studies are needed to further investigate this subject.

### Limitations

Several limitations to this study must be acknowledged. Despite strict control of inclusion and exclusion criteria, our follow-up period, which was set at a minimum of 1 year after surgery and 6 months after AED withdrawal, may not account for the potential increase in seizure recurrence with prolonged observation. In addition, although 116 children were analyzed, the presence of an abnormal EEG in only 29 of them limited our statistical insight. One of the limitations of our study is the rapid tapering protocol of AEDs that we used. Due to this approach, some patients may experience transient withdrawal seizures that do not necessarily indicate long-term seizure recurrence. When interpreting our results, it is important to distinguish between these withdrawal seizures and true seizure recurrence. Another major challenge was retrospectively determining the origin of seizure recurrences, especially when the epileptic focus is not visible on MRI. In addition, some patients quickly returned to a seizure-free state. This rapid resolution made it difficult to determine retrospectively the exact onset of their seizures. Therefore, we could not determine whether the seizure recurrence originated from the resected areas. Finally, future prospective studies should be broader, include different pathological types and lesion sites, and consider a more gradual AED tapering protocol or a clearer distinction between withdrawal seizures and long-term recurrence to elucidate the mechanisms behind seizure recurrence after AED withdrawal.

### Conclusions

In conclusion, AED withdrawal in postoperatively seizure-free children is safe and feasible. A greater number of AEDs used at the time of surgery, presence of IED on postoperative EEG, and incomplete resection predispose to seizure recurrence. The presence of IED on postoperative EEGs indicates a potential incomplete resection of the epileptogenic zone. However, relying on the IED locations to assess the completeness of resection may be misleading. Similarly, while the emergence of IED on postoperative EEG suggests a higher risk of seizure recurrence, the concordance of IED sites with surgical sites does not necessarily imply an increased risk of seizure recurrence after AED discontinuation. For children with abnormal EEG after surgery, the decision to discontinue AED should be made more cautiously, regardless of the relative location of the discharge site and the surgical area. This information will be helpful in exploring the relationship between IED and seizure recurrence and help in making rational decisions on AED withdrawal.

## Data Availability

All data relevant to the study are included in the article or uploaded as supplementary information.
