# Supplementary material for: Postoperative interictal epileptiform discharges predict seizure recurrence after antiepileptic drug withdrawal regardless of concordance with surgical site
Source: World J Pediatr Surg. 2024 Feb 17;7(1):e000641. doi: 10.1136/wjps-2023-000641 (PMC10875540; doi:10.1136/wjps-2023-000641)
Supplement: Supplementary data [file wjps-2023-000641supp001.pdf]

Pathological findings	Location	EEG at 6 months after surgery (normal or IED sites)	AEDs at surgery	AEDs at follow-up
Dysembryoplastic neuroepithelial tumor	left occipital	left parietal+left occipital	levetiracetam	none
FCD IIb	left occipital	normal	Oxcarbazepine	none
Ganglioglioma	right parietal	normal	levetiracetam	none
Ganglioglioma	right frontal	normal	levetiracetam	none
Pilocytic astrocytoma	left temporal	left temporal	Oxcarbazepine	Oxcarbazepine
Dysembryoplastic neuroepithelial tumor	left frontal	normal	Oxcarbazepine	Oxcarbazepine
FCD IIIb	left temporal	normal	Oxcarbazepine	Oxcarbazepine
Cavernous hemangioma	right frontal	normal	Oxcarbazepine	none
Ganglioglioma	right frontal	normal	Oxcarbazepine	none
Papillary glioneuronal tumor	right frontal	normal	Oxcarbazepine	none
Dysembryoplastic neuroepithelial tumor	right frontal+right temporal	normal	levetiracetam	levetiracetam
Ganglioglioma	right frontal+right temporal	normal	levetiracetam	levetiracetam
Dysembryoplastic neuroepithelial tumor	left temporal	right frontal	sodium valproate	none
Papillary glioneuronal tumor	right frontal+right parietal	normal	Oxcarbazepine	none
Dysembryoplastic neuroepithelial tumor	right frontal+right parietal	left parietal	Oxcarbazepine	none
Arachnoid cyst	left temporal	left occipital+left frontal	levetiracetam+Oxcarbazepine	levetiracetam+Topiramate
Pilocytic astrocytoma	right frontal	normal	levetiracetam	levetiracetam
Pilocytic astrocytoma	right frontal	normal	levetiracetam	levetiracetam
Cavernous hemangioma	left temporal	normal	Oxcarbazepine	levetiracetam
FCD IIb	right parietal	bilateral frontal+bilateral temporal+bilateral parieta	Topiramate+Oxcarbazepine+sodium valproate	Oxcarbazepine+Topiramate+sodium valproate
FCD IIa	right parietal	left hemisphere	Oxcarbazepine+sodium valproate	Oxcarbazepine+Topiramate+sodium valproate
FCD IIa	right occipital	normal	sodium valproate+Clonazepam	none
FCD IIb	right occipital	normal	sodium valproate+Oxcarbazepine+Clonazepam	none
Ganglioglioma	left frontal	right frontal+right parietal	Oxcarbazepine	Oxcarbazepine
FCD IIb	left frontal	left parietal+left occipital	Oxcarbazepine	Oxcarbazepine
Pilocytic astrocytoma	right frontal	right frontal+right temporal	Clonazepam+sodium valproate	levetiracetam+Oxcarbazepine+Clonazepam+lamotrigine
Dysembryoplastic neuroepithelial tumor	right temporal	normal	Oxcarbazepine	none
Ganglioglioma	left frontal	left parietal	levetiracetam	levetiracetam
Pilocytic astrocytoma	left frontal	normal	Oxcarbazepine	Oxcarbazepine
Pleomorphic Xanthoastrocytoma	left temporal	normal	Oxcarbazepine	none
Cavernous hemangioma	left frontal+left parietal	left frontal+left parietal	sodium valproate	sodium valproate
FCD IIa	right frontal	left frontal	levetiracetam	levetiracetam
FCD IIb	right frontal	left frontal+left temporal	Oxcarbazepine	Oxcarbazepine
Papillary glioneuronal tumor	left temporal	normal	Oxcarbazepine	none
Pilocytic astrocytoma	left frontal+left temporal	normal	sodium valproate	none
Pilocytic astrocytoma	left frontal	normal	sodium valproate	none
Ganglioglioma	left frontal+left parietal	normal	Oxcarbazepine	none
FCD IIa	right temporal	normal	levetiracetam	none
Papillary glioneuronal tumor	right frontal	normal	sodium valproate	none
Ganglioglioma	right frontal	right frontal+right temporal	Oxcarbazepine	Oxcarbazepine
Dysembryoplastic neuroepithelial tumor	left occipital	normal	levetiracetam	none
FCD IIa	left parietal	normal	levetiracetam	none
Ganglioglioma	left temporal+left frontal	whole brain	sodium valproate	sodium valproate
Cavernous hemangioma	left frontal	normal	sodium valproate	none
vascular malformation	left frontal	normal	sodium valproate	none
Pleomorphic Xanthoastrocytoma	left parietal	normal	sodium valproate	none
Dysembryoplastic neuroepithelial tumor	right frontal	normal	Oxcarbazepine	none
Cavernous hemangioma	left parietal	normal	levetiracetam	none
Cavernous hemangioma	left parietal	normal	Oxcarbazepine	none
Dysembryoplastic neuroepithelial tumor	left parietal	normal	Oxcarbazepine+sodium valproate	none
Papillary glioneuronal tumor	left parietal	normal	Oxcarbazepine+sodium valproate	none
Papillary glioneuronal tumor	right occipital	normal	sodium valproate	sodium valproate
FCD Ia	right frontal	normal	sodium valproate	Oxcarbazepine

FCD IIIb	right frontal	normal	Oxcarbazepine	Oxcarbazepine
Cavernous hemangioma	right frontal+right parietal	normal	Clonazepam+Oxcarbazepine	none
Cavernous hemangioma	right frontal+right parietal	left temporal	Oxcarbazepine+sodium valproate	none
Papillary glioneuronal tumor	left parietal	normal	Oxcarbazepine	none
Ganglioglioma	right temporal	normal	levetiracetam	levetiracetam
encephalomalacia	right frontal	normal	Oxcarbazepine	Oxcarbazepine
vascular malformation	left temporal	right temporal	Oxcarbazepine	Oxcarbazepine
Cavernous hemangioma	right frontal	right frontal+right temporal	levetiracetam	levetiracetam
Diffuse astrocytoma	right parietal	normal	Oxcarbazepine	none
FCD IIIa	right frontal+right parietal	normal	levetiracetam	none
FCD Ib	right frontal+right parietal	normal	Oxcarbazepine	none
Diffuse astrocytoma	right frontal+right parietal	normal	Oxcarbazepine+Clonazepam	Oxcarbazepine+Clonazepam
Ganglioglioma	right parietal	normal	Oxcarbazepine+sodium valproate+Clonazepam	Oxcarbazepine+Clonazepam
Dysembryoplastic neuroepithelial tumor	right temporal	normal	levetiracetam	none
Diffuse astrocytoma	left frontal	left frontal	sodium valproate	none
Papillary glioneuronal tumor	left parietal	left parietal	Oxcarbazepine	none
FCD IIb	right temporal	right temporal	Clonazepam	none
FCD IIb	right frontal	left frontal	sodium valproate	none
Gray matter heterotopia	right parietal	normal	sodium valproate+levetiracetam	sodium valproate+levetiracetam+Oxcarbazepine
Arachnoid cyst	right parietal	normal	Oxcarbazepine+sodium valproate	sodium valproate+levetiracetam+Oxcarbazepine
Pilocytic astrocytoma	left parietal	normal	sodium valproate	none
Papillary glioneuronal tumor	left occipital	normal	Oxcarbazepine	none
Pilocytic astrocytoma	left frontal	normal	Oxcarbazepine	Oxcarbazepine
Pilocytic astrocytoma	left temporal	left temporal	levetiracetam	levetiracetam
Gray matter heterotopia	left parietal	normal	sodium valproate	levetiracetam
Gray matter heterotopia	left parietal	normal	Oxcarbazepine	levetiracetam
FCD IIa	right frontal	normal	Oxcarbazepine+sodium valproate+levetiracetam	Oxcarbazepine+sodium valproate+levetiracetam
Cavernous hemangioma	right temporal+right parieta	normal	Oxcarbazepine	none
FCD IIa	left temporal+left occipital	normal	Oxcarbazepine	none
Cavernous hemangioma	left frontal	right parietal	levetiracetam	Oxcarbazepine
demyelinating lesion	left occipital	left occipital+left temporal	levetiracetam	levetiracetam
tuberous sclerosis	left occipital	left temporal	Oxcarbazepine	Oxcarbazepine
Papillary glioneuronal tumor	right temporal	normal	sodium valproate	none
Diffuse astrocytoma	left parietal	normal	sodium valproate	none
Dysembryoplastic neuroepithelial tumor	left occipital	normal	Oxcarbazepine	none
FCD IIb	left parietal	normal	levetiracetam	none
FCD IIa	left parietal	normal	Oxcarbazepine	none
Pilocytic astrocytoma	right temporal	bilateral frontal	Oxcarbazepine	Oxcarbazepine
Cavernous hemangioma	left parietal	left parietal	sodium valproate	sodium valproate
Arachnoid cyst	left frontal	normal	levetiracetam	none
demyelinating lesion	left frontal	normal	Oxcarbazepine	none
FCD IIa	right occipital	normal	sodium valproate	none
FCD IIb	right occipital	normal	Oxcarbazepine	none
FCD IIa	right temporal	normal	levetiracetam	levetiracetam
Pleomorphic Xanthoastrocytoma	left frontal	normal	Oxcarbazepine+sodium valproate	Oxcarbazepine+sodium valproate
Dysembryoplastic neuroepithelial tumor	left frontal	normal	Oxcarbazepine+sodium valproate	Oxcarbazepine+sodium valproate
vascular malformation	left frontal	normal	Clonazepam+Topiramate	sodium valproate+levetiracetam+lamotrigine
vascular malformation	left temporal	bilateral brain	Oxcarbazepine+levetiracetam	sodium valproate+levetiracetam+lamotrigine
Cavernous hemangioma	left frontal+left temporal	left frontal+left temporal	Oxcarbazepine+sodium valproate	sodium valproate+levetiracetam+lamotrigine
encephalomalacia	left frontal+left temporal	normal	levetiracetam	none
demyelinating lesion	left frontal+left temporal	normal	sodium valproate	none
encephalomalacia	left frontal+left temporal	normal	sodium valproate	none
Gray matter heterotopia	right temporal+right parieta	left frontal	Oxcarbazepine	Oxcarbazepine
FCD IIb	right temporal	normal	Oxcarbazepine+Clonazepam+Topiramate	Oxcarbazepine+Clonazepam+sodium valproate

FCD IIb	right frontal+right temporal	bilateral frontal+right parietal	Oxcarbazepine+Clonazepam+sodium valproate	Oxcarbazepine+Clonazepam+sodium valproate
FCD IIa	right frontal+right temporal	right parietal	Oxcarbazepine+sodium valproate	Oxcarbazepine+Clonazepam+sodium valproate
vascular malformation	left frontal+left parietal	right frontal	sodium valproate	none
FCD IIa	left frontal	left temporal	Oxcarbazepine	Oxcarbazepine+sodium valproate+Topiramate+Clonazep
FCD Ic	left frontal	bilateral brain	Oxcarbazepine	Oxcarbazepine+sodium valproate+Topiramate+Clonazep
FCD IIa	left temporal	normal	sodium valproate	none
FCD IIIc	right parietal	normal	levetiracetam	Oxcarbazepine+sodium valproate
FCD Ic	right parietal	normal	Oxcarbazepine	Oxcarbazepine+sodium valproate
Dysembryoplastic neuroepithelial tumor	right parietal	right parietal	levetiracetam	Oxcarbazepine
Papillary glioneuronal tumor	right parietal	right parietal	Oxcarbazepine	Oxcarbazepine
Cavernous hemangioma	left frontal	normal	sodium valproate	sodium valproate
Cavernous hemangioma	left frontal	left frontal	Oxcarbazepine	Oxcarbazepine
FCD IIb	right frontal+right temporal	right hemisphere	sodium valproate+Clonazepam	Magnesium Valproate+Oxcarbazepine
FCD IIb	right frontal+right temporal	right frontal+right parietal+right temporal	Oxcarbazepine+Clonazepam+Topiramate	Magnesium Valproate+Oxcarbazepine
FCD Ib	right frontal+right temporal	right hemisphere	Oxcarbazepine+sodium valproate	Magnesium Valproate+Oxcarbazepine
vascular malformation	left frontal	normal	levetiracetam	none
vascular malformation	left frontal	normal	sodium valproate	none
FCDIc	right temporal	right parietal	Oxcarbazepine	levetiracetam
FCD IIIb	right frontal	normal	levetiracetam	none
FCD Ia	right frontal	normal	Oxcarbazepine	none
Dysembryoplastic neuroepithelial tumor	right temporal	normal	Oxcarbazepine	none
Cavernous hemangioma	right frontal	right occipital+right frontal	Oxcarbazepine	none
FCD IIIa	left frontal	left temporal	levetiracetam	sodium valproate
FCD Ib	left occipital	left temporal	sodium valproate	sodium valproate
vascular malformation	right frontal	normal	Oxcarbazepine	none
Cavernous hemangioma	right frontal	normal	Oxcarbazepine	none
FCD Ic	right frontal	normal	levetiracetam+sodium valproate	levetiracetam
FCD Ia	right frontal	normal	Oxcarbazepine+sodium valproate	levetiracetam
FCD IIa	right parietal+right occipital	bilateral brain	Oxcarbazepine+Clonazepam+Topiramate	sodium valproate+Oxcarbazepine
FCD IIb	right parietal+right occipital	bilateral temporal	sodium valproate+Oxcarbazepine	sodium valproate+Oxcarbazepine
FCD IIa	left frontal	left frontal	Topiramate+Oxcarbazepine	sodium valproate
FCD Ic	left parietal	normal	Oxcarbazepine+sodium valproate	sodium valproate
Dysembryoplastic neuroepithelial tumor	right parietal	normal	Oxcarbazepine+Clonazepam+sodium valproate	none
Dysembryoplastic neuroepithelial tumor	right parietal	normal	sodium valproate+lamotrigine	none
vascular malformation	right temporal	normal	Oxcarbazepine	Oxcarbazepine
Ganglioglioma	left temporal	bilateral parietal+left temporal	sodium valproate	sodium valproate
Cavernous hemangioma	right frontal+right parietal	normal	Oxcarbazepine	Oxcarbazepine
Cavernous hemangioma	right frontal+right parietal	normal	Oxcarbazepine	Oxcarbazepine
Ganglioglioma	left parietal	normal	sodium valproate+Oxcarbazepine	none
Pilocytic astrocytoma	right frontal	normal	Oxcarbazepine+levetiracetam	none
Dysembryoplastic neuroepithelial tumor	right frontal	normal	Oxcarbazepine+sodium valproate	none
tuberous sclerosis	right frontal+right parietal	normal	Clonazepam+Oxcarbazepine	none
Gray matter heterotopia	right frontal+right parietal	normal	sodium valproate+lamotrigine	none
FCD IIIb+Dysembryoplastic neuroepithelia	left temporal	normal	levetiracetam	none
FCD IIb	right parietal+right occipital	normal	levetiracetam	levetiracetam
FCD IIa	right parietal+right occipital	normal	sodium valproate	levetiracetam
vascular malformation	left parietal	normal	Oxcarbazepine	none
Cavernous hemangioma	right temporal	normal	levetiracetam	levetiracetam
FCD Ia	left temporal+left occipital	normal	Oxcarbazepine	none
Gray matter heterotopia	left frontal	left frontal	sodium valproate+Oxcarbazepine	Oxcarbazepine
tuberous sclerosis	left parietal	left parietal	Oxcarbazepine+levetiracetam	Oxcarbazepine
tuberous sclerosis	right frontal+right temporal	left frontal+left occipital	levetiracetam+Clonazepam	levetiracetam+Topiramate+Clonazepam
encephalomalacia	right frontal+right temporal	left temporal+left occipital	levetiracetam+Topiramate+Clonazepam	levetiracetam+Topiramate+Clonazepam
Dysembryoplastic neuroepithelial tumor	right frontal+right temporal	left temporal	Oxcarbazepine+levetiracetam	levetiracetam+Topiramate+Clonazepam

Ganglioglioma	right parietal	normal	levetiracetam	none
Diffuse astrocytoma	right parietal	normal	sodium valproate	none
Dysembryoplastic neuroepithelial tumor	left temporal	normal	sodium valproate	sodium valproate
demyelinating lesion	right occipital	normal	levetiracetam	none
encephalomalacia	right occipital	normal	Oxcarbazepine	none
vascular malformation	right frontal+right temporal	left temporal	sodium valproate	none
Cavernous hemangioma	right frontal+right temporal	normal	Oxcarbazepine	none
Ganglioglioma	right frontal+right parietal	normal	levetiracetam	none
Ganglioglioma	right frontal+right parietal	normal	Oxcarbazepine	none
Ganglioglioma	right frontal+right temporal	right frontal+right temporal+right parietal	levetiracetam	sodium valproate
Ganglioglioma	right temporal	normal	Oxcarbazepine	none
Pilocytic astrocytoma	left temporal	normal	Oxcarbazepine	none
vascular malformation	right frontal	right frontal	levetiracetam	Oxcarbazepine
Cavernous hemangioma	right parietal	right parietal	Oxcarbazepine	Oxcarbazepine
Dysembryoplastic neuroepithelial tumor	left temporal	normal	Oxcarbazepine	levetiracetam
Cavernous hemangioma	right frontal	normal	Oxcarbazepine	none
Dysembryoplastic neuroepithelial tumor	right parietal	left temporal	sodium valproate	none
Pilocytic astrocytoma	right parietal	bilateral frontal	Oxcarbazepine	none
Dysembryoplastic neuroepithelial tumor	left parietal	normal	sodium valproate	levetiracetam
Pilocytic astrocytoma	left parietal	normal	Oxcarbazepine	levetiracetam
encephalomalacia	right temporal+right parieta	normal	sodium valproate+levetiracetam	Oxcarbazepine

BMJ Publishing Group Limited (BMJ) disclaims all liability and responsibility arising from any reliance placed on this supplemental material which has been supplied by the author(s)

am
am
